# Diet, oxidative stress and MAFLD: a mini review

**DOI:** 10.3389/fnut.2025.1539578

**Published:** 2025-03-04

**Authors:** Zenan Hu, Hanxun Yue, Na Jiang, Liang Qiao

**Affiliations:** ^1^The First School of Clinical Medicine, Lanzhou University, Lanzhou, China; ^2^Department of Gastroenterology, The First Hospital of Lanzhou University, Lanzhou, China; ^3^School of Public Health, Lanzhou University, Lanzhou, China; ^4^Storr Liver Centre, Westmead Institute for Medical Research, The University of Sydney and Westmead Hospital, Westmead, NSW, Australia

**Keywords:** diet, oxidative stress, MAFLD, NAFLD, MASLD

## Abstract

Globally, metabolic dysfunction-associated fatty liver disease (MAFLD), also known as non-alcoholic fatty liver disease (NAFLD) or metabolic dysfunction-associated steatotic liver disease (MASLD), is a common chronic liver disease. The progression of MAFLD leads to a vicious cycle in which oxidative stress results from the disease that is augmenting de-novo lipid levels and increases steatosis. Most non-enzymatic antioxidants are present in food. Therefore, the present review summarizes the findings of studies on food-derived antioxidants and presents an oxidative stress-related regulatory network in MAFLD, offering new ideas for MAFLD prevention and treatment.

## 1 Introduction

Metabolic dysfunction-associated fatty liver disease (MAFLD) is characterized by the presence of fat in ≥5% hepatocytes, independent of excess alcohol consumption and other chronic liver diseases (1, 2). Previously referred to as non-alcoholic fatty liver disease (NAFLD), the term MAFLD was introduced to encompass the metabolic factors driving this disorder (3). Some experts alternatively refer to it as metabolic dysfunction-associated steatotic liver disease (MASLD) (4, 5). For consistency, this review refers to the condition as MAFLD.

MAFLD primarily arises from the accumulation of lipids in hepatic cells and presents as a spectrum of conditions ranging from simple steatosis to steatohepatitis. This disease can be classified into two subtypes: metabolic dysfunction-associated fatty liver (MAFL), marked by hepatic steatosis without significant liver injury; metabolic dysfunction-associated steatohepatitis (MASH), characterized by hepatocyte damage, inflammatory cell infiltration, and hepatic cell death (6). Although often clinically perceived benign, MAFLD can advance to severe liver fibrosis, ultimately resulting in cirrhosis or hepatocellular carcinoma (HCC). During this progression, lipid accumulation induces mitochondrial dysfunction and oxidative stress, leading to hepacyte damage (7, 8). From 2016 to 2019, the global prevalence of MAFLD rose from 25% to approximately 30%, with the mortality rate increasing from 0.77/1000 to 1.65/1000 person-years, underscoring its significance as a leading cause of chronic liver disease-related morbidity and mortality worldwide (9). Moreover, MAFLD is strongly associated with metabolic syndrome, type 2 diabetes mellitus, atherosclerotic cardiovascular disease, and colorectal neoplasms (10, 11).

A central mechanism driving MAFLD progression is oxidative stress, which results from an imbalance between the production and elimination of reactive oxygen species (ROS) (12–15). This imbalance leads to accumulations of ROS, directly injuring hepatocytes and producing toxic metabolites like malondialdehyde (1). Moreover, oxidative stress creates a feedback loop that exacerbates lipid accumulation and steatosis, further amplifying MAFLD progression (16). The severity of MAFLD correlates with oxidative stress levels and oxidative stress is a potential diagnostic marker for MAFLD (17).

Extensive research has explored the mechanism of oxidative stress inducing MAFLD (18). Early work by Maurizio and Novo (19) identified the role of *Nrf1* in linking oxidative stress to MAFLD by demonstrating that *Nrf1*-induced *CYP4A* upregulation increases ROS generation and inhibits antioxidant activity through suppression of *ARE* expression. Similarly, *AHR* has been implicated in MAFLD pathogenesis through its regulation of *CYP1A1*, influencing oxidative stress pathways (20). Recent studies have highlighted the role of genetic factors, such as *SIRT5* rs12216101 T > G, in amplifying oxidative stress in MAFLD patients (21). Additionally, several miRNAs and ncRNAs have been recognized as crucial regulator of oxidative stress in MAFLD (22–24). Until now, *Nrf2* emerges as a key molecule in modulating oxidative stress in MAFLD (25).

Dietary and lifestyle factors play a pivotal role in MAFLD progression (26–28). The oxidative balance score (OBS), which integrates pro- and antioxidant components from diet and lifestyle, has been shown to reflect the overall oxidative stress burden. Higher OBS was significantly associated with a lower risk of MAFLD (29). Real-world OBS analyses demonstrate that adopting a healthy diet and lifestyle, independently or in combination, can mitigate oxidative stress and significantly lower MAFLD onset and development (30). However, the precise role of diet in linking oxidative stress to MAFLD remains inadequately understood. We reviewed studies on food-derived antioxidants from the past decade, summarized representative research, and depicted a regulatory network targeting oxidative stress, centered on *Nrf2* and mediated by endogenous molecules and signaling pathways. This review examined the impact of diet on oxidative stress, providing insights into potential preventive and therapeutic strategies for MAFLD.

## 2 Fruits, vegetables, grains, and herbs play a major role in alleviating oxidative stress

Many substances in fruits, vegetables, and grains can directly or indirectly inhibit MAFLD progression by suppressing oxidative stress. According to Li et al. (31), hesperetin, a flavonoid present in citrus fruits, boosted the antioxidant activity by triggering the *PI3K*/*Akt* pathway and reduced ROS overproduction by activating the *Nrf2* pathway during MAFLD progression. Fan et al. reported that nobiletin, a polymethoxylated flavone primarily extracted from citrus peels, accelerated the dissociation of the *Keap1*-*Nrf2* complex and promoted *Nrf2* nuclear translocation, thereby alleviating MAFLD (32). In another study, total flavonoids extracted from *Citrus changshan-huyou* were reported to alleviate oxidative stress in MAFLD by upregulating *miR-137-3p* expression, which subsequently downregulated *NOXA2*/*NOX2*, reducing ROS generation (33). Found abundantly in apples, phloretin (a dihydrochalcone phenolic compound), alleviated oxidative stress by regulating the *ERK*/*Nrf2* pathway, which enhanced the antioxidant response (34, 35). Extracted from blueberries and grapes, pterostilbene was showed to alleviate oxidative stress and enhance fatty acid metabolism and decomposition via activation of the *AMPK*/*mTOR* pathway in hepatocytes (36). *Euterpe oleracea* Mart, popularly known as açai, is a palm tree fruit usually found in the Brazilian Amazonas and Pará states. The aqueous extract of açai (AAE) significantly prevented oxidative stress in patients with MAFLD (37).

Apigenin, a naturally occurring flavonoid in various fruits and leafy vegetables, activated the *Nrf2* signaling pathway to reduce oxidative stress, thereby attenuating MAFLD (38). Liensinine is an isoquinoline alkaloid commonly found in *Nelumbo nucifera Gaertn* (lotus seeds) which is often consumed in Asia. Liensinine was shown to inhibit oxidative stress by upregulating *Nrf2* and modulating the *AMPK* signaling pathway by *TAK1* activation (39). Oligosaccharides, extracted from *Porphyra yezoensis* (a commonly consumed algea in East Asia), alleviated oxidative stress by downregulating the *TGF-β* signaling pathway, which is implicated in liver inflammation and fibrosis (40).

A major flavonoid found in buckwheat, rutin alleviated oxidative stress in diabetes-associated MAFLD through the *AMPK* signaling pathway, which plays a crucial role in energy homeostasis and stress response (41, 42). Betaine is commonly found not only in beets and whole grains but also in shrimps and shellfish. It could regulate lipid metabolism and mitochondrial function as well as inhibit oxidative stress, making it a promising candidate for MAFLD prevention and treatment (43).

Some traditional Chinese herbs may be used as condiments in food. Aescin, a bioactive compound derived from the ripe dried fruits of *Aesculus chinensis Bunge*, ameliorated oxidative stress, thus exerting a curative impact on MAFLD. The mechanism underlying aescin’s action was that it interacted with *Keap1*, leading to an enhanced translocation of *Nrf2* into the nucleus (44). Alpinetin is a novel plant flavonoid isolated from *Alpinia katsumadai Hayata*, which inhibited oxidative stress by enhancing *SOD1*/*HO-1*/*Nrf2* expression in MAFLD (45). Safranal is the active constituent of saffron (B.O.: *Crocus sativus*). Sabir et al. demonstrated that safranal treatment reduced the levels of oxidative stress indicators in MAFLD animal models (46). Rhamnetin extracted from *Rhamnus davurica* Pall exhibited antioxidative properties, which were effective against steatohepatitis and hepatocellular carcinoma (47).

## 3 Animal foods and supplements have inhibitory effects on oxidative stress

Astaxanthin presents in shrimp, crab, salmon, algae, and other marine organisms. As a carotenoid, astaxanthin works by neutralizing ROS and reducing oxidative stress (48). Wu et al. (49) found that astaxanthin attenuated mitochondrial dysfunction by upregulating *FGF21*/*PGC-1α*, thus alleviating oxidative stress in MAFLD (50). Moreover, astaxanthin has been proved to more advantageous than vitamin E in reversing steatohepatitis (51). Omega-3 fatty acids exist in several forms and are abundantly present in oily fish (26). According to a systematic review, omega-3 polyunsaturated fatty acids were effective in counteracting oxidative stress in early-stage MAFLD (52).

López-Oliva et al. (53) showed that *α*-lactalbumin, found in dairy products, induced oxidative stress by upregulating *XRαβ*/*SREBP-1-c*/*PPARγ* expression and diminishing *PPARα*/*CPT-1* expression and *AMPKα* phosphorylation. However, Chen et al. (54) reported that the *α*-lactalbumin peptide Asp-Gln-Trp (DQW) might serve as an effective dietary supplement for alleviating MAFLD by reducing oxidative stress (53). Additionally, the α-lactalbumin peptide Gly-Ile-Asn-Tyr (GINY) alleviated oxidative stress in MAFLD progression (55). As bacteriological studies have advanced, probiotics have been found to inhibit oxidative stress in MAFLD (56). *Lactobacillus rhamnosus* GG, a probiotic frequently found in dairy products such as cheese, inhibited oxidative stress by activating the *Nrf2* pathway in MAFLD (57–59).

In addition to omega-3, oleoylethanolamide supplements exhibited an excellent ability to inhibit oxidative stress in MAFLD progression (60). Giudetti et al. (61) reported that oleoylethanolamide regulated *Nrf1* and *Nrf2* differently, which increased *Nrf1* levels but decreased *Nrf2* levels. Reda et al. (62) elaborated that vitamin D3 inhibited oxidative stress in MAFLD by reducing *SREBP-1-c* expression and increasing *PPARα* expression to activate the *NF-κB* signaling pathway.

## 4 Beverages and snacks are also sources of antioxidants

Green tea is a popular traditional Chinese drink, which rich in catechins, particularly epigallocatechin gallate (EGCG). EGCG has been shown to reduce oxidative stress-induced progression of MAFLD by regulating the *Nrf2*, *AMPK*, *SIRT1*, *NF-κB*, *TLR4*/*MYD88*, *TGF-β*/*SMAD*, and *PI3K*/*Akt*/*FoxO1* signaling pathways (63). Zhou et al. (64) demonstrated that some bioactive flavor compounds present in alcoholic beverages, such as xanthohumol, resveratrol, quercetin, anthocyanins, tetramethylpyrazine, and terpenes, could alleviate oxidative stress. “Baijiu,” the most common spirit in China, is more beneficial than beer and wine in alleviating MAFLD. Since alcohol is a recognized Group 1 carcinogen, we do not recommend consuming alcoholic beverages, especially spirits.

Moreover, Loffredo et al. (65) observed that coca polyphenols suppressed oxidative stress by downregulating *NOX2* expression, suggesting that dark chocolate produces antioxidant effects in patients with steatohepatitis. Carminic acid is frequently used as a colorant in beverages and snacks, which could mitigate oxidative stress by blocking the *TNF-α* pathway and activating the *Nrf2* pathway (66).

## 5 Metal elements bidirectionally regulates oxidative stress

The intake of different metal elements has varying effects on oxidative stress-induced MAFLD progression. On analyzing numerous studies *in vivo*, Xu et al. (67) discovered that selenium reduced steatosis and fibrosis in MAFLD by alleviating oxidative stress. Zhong et al. (68) reported that excessive copper accumulation induced oxidative stress and lipogenesis, while inhibiting lipolysis. They also elucidated that copper-induced oxidative stress promoted *Nrf2* recruitment to the *PPARγ* promoter and improved lipogenesis, providing evidence for *Nrf2* as a potential therapeutic target for MAFLD (68). Iron overload also induced oxidative stress, thereby damaging hepatocytes. *Caveolin-1* overexpression augmented the iron storage capacity of hepatocytes by activating the ferritin light chain/ferritin heavy chain pathway in MAFLD and subsequently alleviating excess ferrous ion-induced oxidative stress in the liver (69, 70). Silver nanoparticles significantly elevated oxidative stress levels in mice with MAFLD (71).

## 6 Discussion

**Figure 1:**
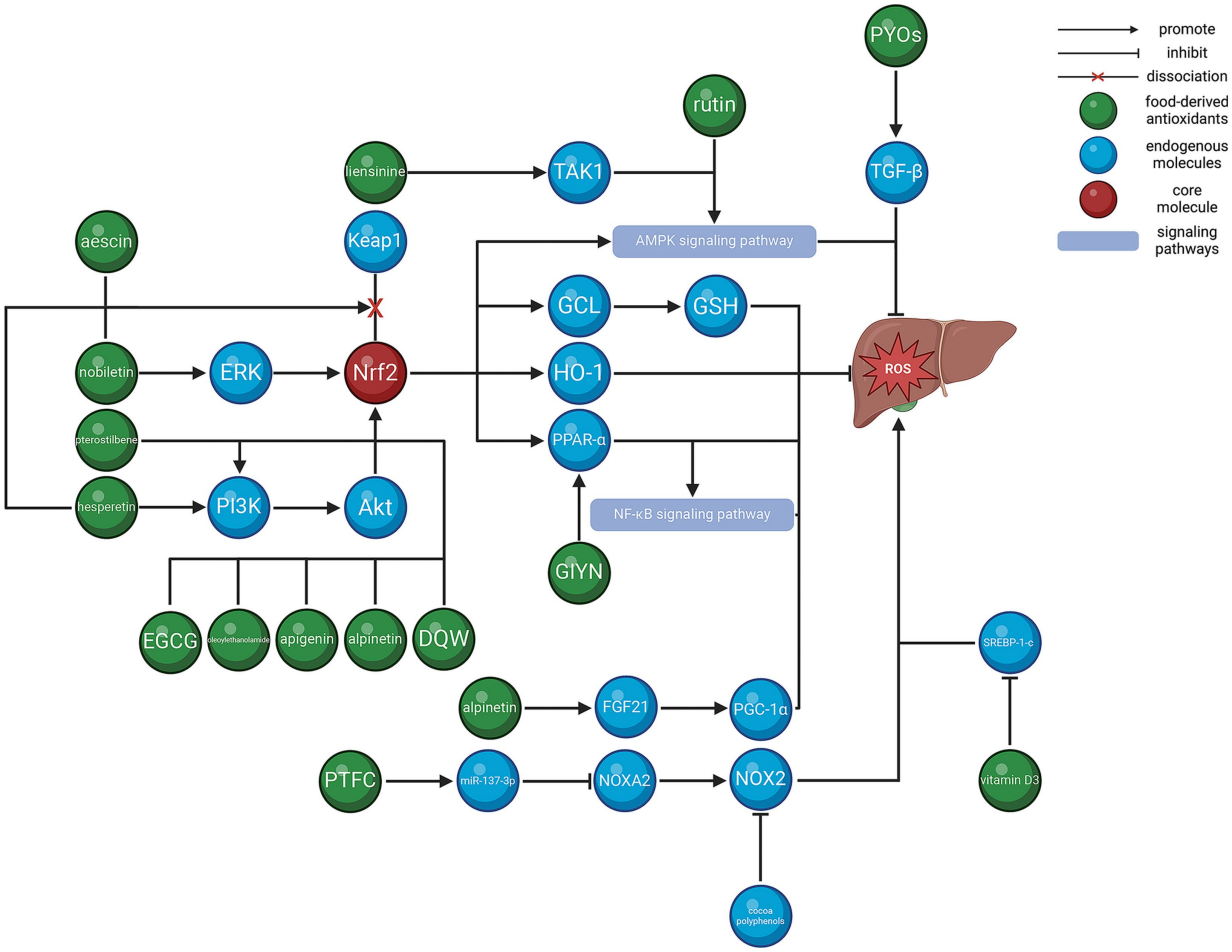
Regulatory network of food-derived antioxidants in oxidative stress in hepatocytes.

This review highlights studies on food-derived antioxidants and the role of diet in the oxidative stress pathway that contributes MAFLD, with the aim of clarifying the regulatory network involved (Table 1 and Figure 1). Based on the findings, we proposed that a healthy diet can alleviate MAFLD by reducing oxidative stress.

**Table 1:** Food-derived antioxidants, models, exposure and related endogenous molecules.

Antioxidant	Sources	Models (treatment)	Related mol.	Ref.
Hesperetin	Citrus fruit	HepG2 cell line (2.5, 5, or 10 μM for 24 h) Rats (100 or 300 mg/kg/d for 16w)	*Nrf2*, *PI3K*, *Akt*, *Keap1*	(31)
Nobiletin	Citrus peel	L02 and HepG2 cell lines (5, 10, or 25 μM for 24 h) mice (200 or 500 mg/kg/d for 4w)	*Nrf2*, *Keap1*	(32)
PTFC	*Citrus changshan-huyou*	AML-12 cell line (5, 10, or 20 μM for 24 h) mice (25 or 50 mg/kg/d for 12w)	*miR-137-3p*, *NOXA2*, *NOX2*	(33)
Phloretin	Apple fruit	Huh-7 cell line (50, 100, or 150 μM for 24 h) Mice (50, 100, or 200 mg/kg/d for 16w) rats (30 mg/kg/d for 5d)	*Nrf2*, *ERK*, *HO-1*, *GCL*, *GSH*	(34, 35)
Pterostilbene	Blueberry, grape	HepG2 cell line (12.5, 25, 50, 100, or 200 μM for 1 h) Mice (30, 45 or 60 mg/kg for 24 h)	*Nrf2*, *HO-1*, *PPAR-α*, *AMPKs*	(36)
AAE	*Euterpe oleracea* Mart	HepG2 cell line (12.5, 25, 50, 100, 200, or 400 μM) Mice (3 g/kg/d for 12w)	*-*	(37)
Apigenin	Fruits and leafy vegetables	Hepa1-6 cell line (0.2–64 μM for 24 h) Mice (30 mg/kg/d for 3w)	*Nrf2*	(38)
Liensinine	*Nelumbo nucifera Gaertn*	L02 and AML-12 cell lines (1.25, 2.5, 5, 10, 20, 30, 40, 50, or 60 μM for 96 h) Mice (15, 30, or 60 mg/kg/d for 16w)	*TAK1*, *AMPKs*	(39)
PYOs	*Porphyra yezoensis*	Mice (100 or 225 mg/kg/d for 6w)	*AMPKs*	(40)
rutin	Buckwheat	Mice (100 or 200 mg/kg/d for 8w)	*AMPKs*	(41)
betaine	Beet, whole grain, shrimp, shellfish	–	*–*	(43)
Aescin	*Aesculus chinensis Bunge*	HepG2 cell line (2 μM for 24 h) Mice (free access to food)	*Nrf2*	(44)
Alpinetin	*Alpinia katsumadai Hayata*	HL-7702 cell lines (20, 40, or 80 μM for 24 h) Mice (12.5, 25 or 50 mg/kg/d for 16w)	*Nrf2*, *SOD1*, *HO-1*	(45)
Safranal	*Saffron*	Rats (250 or 500 mg/kg/d for 4w)	*–*	(46)
Rhamnetin	*Rhamnus davurica* Pall	HepG2 cell line (100, 500, 600, or 1,000 μM for 24 h)	*–*	(47)
Astaxanthin	Shrimp, crab, salmon, algea	L02 cell line (30, 60, or 90 μM for 24 h) Mice (10, 30 or 60 mg/kg/2d for 10w) Mice (0.02% of food for 10w)	*FGF21*, *PGC-1α*	(49, 51)
Οmega-3	Oily fish	–	*–*	(60)
DQW	Dairy products	HepG2 cell line (150 mg/mL for 24 h) Mice (30 or 60 mg/kg/d for 12w)	*Nrf2*, *PPARα*, *HO-1*	(54, 55)
GINY	Dairy products	HepG2 cell line (250 mg/mL for 24 h)	*PPARα*	(55)
Oleoylethanolamide	Supplement	Huh-7 cell line (10 μM for 24 h) Rats (10 mg/kg/d for 2w)	*Nrf2*	(60, 61)
Vitamin D3	Supplement	Rats (1,000 IU/kg 3d/w for 10w)	*SREBP-1-c*, *PPARα*	(62)
EGCG	Green tea	–	*Nrf2*, *AMPKs*, *SIRT1*	(63)
Cocoa polyphenols	Dark chocolate	–	*NOX2*	(65)
Selenium	Nuts, game meat	–	*–*	(67)

This review primarily includes *in vitro* and *in vivo* studies, along with a small number of randomized controlled trials (RCTs). In the *in vitro* studies included in this review, the cell lines used include human normal liver cell lines (L02, HL-7702), human HCC cell lines (HepG2, Huh-7), mouse normal liver cell line (AML-12), and mouse HCC cell line (Hepa1-6). Included in *in vivo* experiments primarily used mice and rats as animal models, with oral gavage being the most common administration method, although some studies also employed intraperitoneal injection. The dosage, exposure methods, and exposure duration are presented in Table 1.

Non-enzymatic antioxidants, predominantly found in plant-based foods, are key contributors to oxidative stress mitigation (1). Citrus fruits, for instance, are particularly effective in preventing and managing MAFLD due to their high flavonoids content. Similarly, common fruits like apples, grapes, and, blueberries play a vital role in reducing oxidative stress. Rare fruits, such as açai, are recommended in regions with a high prevalence of MAFLD, given their potent antioxidant properties.

Vegetables, including certain varieties unique in Asia, also demonstrate benefits for MAFLD management, suggesting the value of sharing dietary practices across regions. For example, the Mediterranean diet comprises nutrients and compounds with antioxidant properties, such as polyphenols, carotenoids, fiber, polyunsaturated fatty acids, low-refined foods, and low-sugar foods. In addition, herbal medicine has been identified as a promising therapeutic option for addressing oxidative stress in MAFLD (72–74).

While animal-based foods also contain non-enzymatic antioxidants, excessive consumption, particularly of meat, increases the risk of MAFLD (75). Alternatively, antioxidants derived from animal resources can be provided as supplements. Although certain alcoholic beverages may possess antioxidant properties, their consumption is not recommended for patients with MAFLD due to the potential for liver damage. Selenium, an essential trace element, can be obtained either from selenium-rich foods, such as nuts and game meat, or from carefully selected supplements (76). However, it is critical to avoid excessive intake of mental elements, which may exacerbate MAFLD. Additionally, food contamination remains a significant factor that can amplify the adverse effects of oxidative stress on MAFLD (77).

It is evident that *Nrf2* plays a central role in combating oxidative stress in MAFLD. Majority of food-derived antioxidants (e.g., aescin, nobiletin, pterostilbene, hesperetin, and EGCE) can upregulate or activate *Nrf2* directly or indirectly. *Nrf2* can inhibit oxidative stress by activating multiple regulatory axes and signaling pathways (e.g., MAPK and NF-κB). Therefore, centered on *Nrf2*, food-derived antioxidants and endogenous molecules form a regulatory network for oxidative stress.

Some of the studies included in this review were conducted in the context of a high-fat diet. Notably, AAE exhibits superior antioxidant capacity in the context of a high-fat diet, suggesting a potential interaction between food-derived antioxidants and dietary composition. This highlights the need for synchronizing antioxidant intake with dietary adjustments. Furthermore, the relationship among diet, oxidative stress, and MAFLD should be explored within a holistic and dynamic framework.

This study has limitations, as it primarily focuses on *in vitro* and *in vivo* research, with limited inclusion of RCTs the test these theories in the real world. Moreover, lifestyle modifications, such as physical exercise, play a crucial role in regulating oxidative stress in MAFLD (78, 79). Future research should explore the synergistic effects of diet and lifestyle interventions on oxidative stress, along with the underlying mechanisms. Additionally, more RCTs are needed to validate these findings and provide stronger evidence for clinical application.
